# Current challenges in teaching HAIs-PC in nursing education in Vietnam and Cambodia: a qualitative study

**DOI:** 10.12688/f1000research.139734.1

**Published:** 2023-11-30

**Authors:** Anh Tuan Truong, PrevInf Group: Capacitating Asia's Nursing Students on Innovative and Sustainable Prevention and Control of Healthcare-associated Infections

**Affiliations:** 1 Nursing, Nam Dinh University of Nursing, Nam Dinh city, Nam Dinh Province, 420000, Vietnam

**Keywords:** healthcare associated infections prevention and control, nursing education, challenges, Vietnam and Cambodia.

## Abstract

**Background**: There is an insufficient understanding of factors that impede nursing students’ learning of healthcare-associated infection prevention and control in developing countries. This study aimed to explore current challenges in healthcare-associated infection control and prevention education in the nursing curriculum in two Vietnamese and two Cambodian universities.

**Methods**: Exploratory research was conducted through consultation of education programs and a qualitative study design utilizing interviews and focus group discussions. Data collection was conducted through interviews with university board members and focus group discussions with lecturers and tutors. The data were analyzed by using content analysis methods.

**Results:** The research results indicated that there were three generic themes of challenges in teaching HAIs-PC in nursing education in Vietnam and Cambodia. They were Implementation of healthcare associated infections prevention and control education into nursing curriculum, Positive aspects fostering healthcare associated infections prevention and control learning, Negative aspects hindering healthcare associated infections prevention and control learning.

**Conclusions:** The study results provided evidence of challenges in healthcare associated infections prevention and control education in some Asia higher education institutions. To improve professional safety, universities should pay more attention to developing appropriate teaching methods for healthcare-associated infections prevention and control education to improve students’ practice outcomes.

## Introduction

Healthcare-associated infections (HAIs) are infections people get while receiving health care in any healthcare facility, including hospitals, ambulatory surgical centers, and long-term care facilities. ^1^ Bacteria, fungi, viruses or other, less common pathogens can cause HAIs. HAIs are often acquired from hospitals after 48 hours or more of admission or within 30 days after having received health care. The World Health Organization (WHO) report shows a high rate of Antimicrobial Resistance and estimates 10 million deaths per year due to HAIs in 2050. ^2^ The various consequences of HAIs for patients and the health system are included e.g., the increased morbidity and mortality of patients, prolonged average hospital stay from 7 to 15 days, and increased use of antibiotics leading to a resistance of microorganisms.

The incidence of HAIs is higher in developing countries compared to developed countries. ^2^^,^^3^ Therefore, the increasing prevalence of HAIs in Asian countries increases the costs of healthcare organizations and the morbidity and mortality of patients in already low-income countries. ^4^ The overall prevalence of HAIs in developing countries is around 17.0%, in which the rate of infections acquired in intensive care units is at least two to three times higher than in high-income countries. ^5^

In Vietnam, previous findings indicated that the rate of hospital infections was around 6%, of which respiratory tract infections accounted for the highest rate at 56%. The risk factors associated with HAIs such as age (18-30 years old), gender (male), length of hospital stay (9 days), and invasive procedures. ^6^ The prevalence of HAIs in the adult ICU accounted for around 23-30%. ^7^^,^^8^ In addition, the rate of antibiotic resistance in Vietnam is a significant concern. Tran and colleagues (2019) indicated that the rate of gram-negative bacteria infection in inpatients is high. Infectious diseases accounted for 25% of hospitalizations and had a high rate of mortality (12%). ^9^

On the other hand, in Cambodia, the most-reported ones were respiratory tract infections accounting for 53%. ^10^ The study conducted at the National Hospital for Children indicated that HAIs incidence was around 14%, and the incidence of HAIs in ICU was 50%. ^10^ The various rates of HAIs in different Asian countries and hospitals could be due to different morbidity patterns, treatment protocols, level of development of health systems, or HAIs prevention and control (HAIs-PC). Moreover, the different operational definitions of HAIs adopted in the studies and the low notification and monitoring of these events can also be the reason for the variation in HAIs rates across countries. ^11^

Even though prevalence statistics regarding HAIs are not sufficiently reported, there are many problems, which hinder systematic HAIs-PC work in developing countries. ^12^ The awareness of the healthcare workers is not adequate, and at the same time, the lack of support resources such as education and medical equipment leads to a high percentage of HAIs. In Vietnam, according to Ngo (2018), healthcare workers reported challenges related to HAIs including poor resources, awareness, and patient overload. ^6^ In 2015, WHO emphasized the importance of strengthening HAIs-PC by improving awareness among healthcare workers and patients. ^13^ Governments of different countries indicated that it is necessary to promote healthcare, thereby consolidating and perfecting the system of surveillance, warning, and proactive disease prevention; establishing units of disease control and prevention. ^13^

The provision of quality education is essential for the training and development of the appropriate competencies, knowledge, and skills of healthcare workers, which enables them to provide safe and quality healthcare. In Asian countries, healthcare universities gradually introduced HAIs-PC education programs for students at all levels. ^14^ These programs demonstrated certain effectiveness of education in raising awareness and reducing the rate of HAIs. In Cambodia, following the WHO’s global action plan, efforts have been made to enhance the quality of Cambodia's health institutions’ conditions, including infection prevention and control practices. ^13^ Moreover, the research evidence recommended that hospitals and healthcare universities should provide appropriate HAIs-PC education to students, patients, and healthcare providers, especially, nursing students at the very beginning of their bachelor program. ^15^^,^^16^

In Asian countries, the HAIs-PC education program currently differs between universities and countries in contents and form of practice training, as well as in assessment and recognition. ^27^ These disparities have led to difficulties in evaluating the effectiveness of the education program in improving nursing students’ knowledge and competence in this field. Moreover, there is insufficient scientific research to evaluate the perception of lecturers and learners about HAIs-PC education in developing countries. The research aim thus was to explore current challenges in HAIs-PC education in nursing curriculum in two Vietnamese and two Cambodian universities.

## Methods

### Research design

A qualitative study was conducted through interviews and focus group discussions among managers, nursing faculty teachers, and clinical tutors.

### Settings of study

The study was conducted in four Higher Education Institutions (HEIs) in Vietnam and Cambodia. These institutions offer nursing education at levels of an associate degree in nursing (ADN), Bachelor of Nursing Science (BNS), and Master of Nursing Science (MNS).

### Participants

The participants of the study were invited based on the inclusion criteria: (1) working in the university or in the hospital and teaching contents related to HAIs-PC; (2) having at least a bachelor’s degree; (3) having at least five years’ experience in teaching contents related to HAIs-PC. In each institution, the participants of the study were divided into three groups. The first group consisted of managers, coordinators of scientific boards, and the heads of the nursing department. The second group consisted of nursing faculty teachers who directly teach module of HAIs-PC to nursing students. The third group consisted of clinical tutors who provided instructions for students in the hospitals. They were full-time faculty teachers in the universities or full-time nurses in the hospitals.

### Data collection instruments

There were four instruments used for data collection developed by the research team, including one quantitative and three qualitative instruments.

**A 26-item record collection instrument** was used to collect the indicators of HAIs-PC education in each institution about the overall duration of the specific curriculum, the number of credits for each module including theory and practice, and the lecturer’s and the student’s information in the nursing curriculum. Especially, the HAIs-PC contents and structure in the nursing curriculum were conducted in the course syllabus. To collect data related to the education program, the researchers accessed the HAIs-PC module in the curriculum which is published on the website of the institution.

The qualitative instruments were used to guide the interview of managers and group discussion of teachers and clinical tutors. **The University Board interview guide** explored the perceptions of the participants from each institution, on the HAIs-PC program in their nursing curriculum with guiding points of (1) educational opportunities, (2) HAIs-PC contents in the nursing course, (3) the duration of the module, (4) used active teaching and learning methods, (5) institutional actions for the improvement, (6) students’ learning opportunities, and (7) students’ learning outcomes. **The lecturer focus group topic guide** explored the perception of the nursing faculties for (1) the HAIs-PC contents; (2) the study plan, (3) the continuity between the curricular units, (4) the concept of students’ entrepreneurial skills, (4) institution effort for students and teachers’ competencies, (5) equipment, technologies, or teaching materials, (6) effective learning methods, (7) theoretical references, (8) the teaching process. **The tutor focus group topic guide** explored the perception of the participants on the HAIs-PC program with guiding points of (1) awareness of the nursing students on HAIs-PC, (2) institution support, (3) active learning methods, knowledge gap, (4) students’ challenges and solutions, (5) students’ learning outcomes and (6) hospital and university relationship. The interview and focus group guides were developed by the English-speaking researcher for use in several countries and then collaboratively refined and professionally translated for cross-language use in the local Vietnamese and Cambodian context and culture.

The translated instruments were validated by five lecturers who met the same criteria as the prospective participants. The lecturers were asked to evaluate individual items on the instruments as well as the entire instruments. The primary investigator and a research assistant then revised the contents of the instruments following the lecturers’ comments and suggestions. The two research assistants in each HEI were nursing teachers who taught nursing students related to IPC. They were trained to collect data using the four research instruments by the primary investigator.

All the above instruments can be found in the Extended data.

### Ethical consideration

Preceding the data collection, the study was approved by the Health Sciences Research Unit: Nursing (UICISA: E) of the Nursing School of Coimbra (Portugal) with the reference number P761-3/2021; Ethic Committee of Biomedical Research at the Nam Dinh University of Nursing with the approval number 831/GCN-HĐĐĐ (April 22, 2021); Ethical Review Board for Biomedical Research, Hai Duong Medical Technical University with the approval number 191/QD-DHKTYTHD (March 26, 2021); Ethics Committee at the Bolyno Institute (April 22, 2021); and IU Ethics Committee at the International University (April 22, 2021). The acceptance for data collection was obtained from the Asian partner universities. Study information was provided in Vietnamese and Cambodian, and participants were encouraged to ask questions before they signed the informed consent. The quantitative data collection and the face-to-face interviews and discussion groups were conducted in April, 2021 at the campus or at the hospital premises. The participants were informed about the study and its objectives, as well as the voluntary nature of participation. Alphanumeric characters were used instead of participant’s names in the analysis and publication.

### Data collection

In order to collect the indicators related to the HAIs-PC education program, the research team used data available from the curriculum information at the universities. Moreover, the perception of HAIs-PC education was collected through in-depth interviews and focus group discussions. At the beginning of the focus group session and interviews, explanations about the research goals and regulations were given to the participants. The interviews and group discussions took about 30 to 60 minutes in duration. All interviews and group discussions were recorded via tape recorders. Immediately after each interview or group discussion, the data analysis was conducted to ensure that data saturation was reached. The location and time of the interviews were selected based on the participants’ preferences. After holding interviews, in case of any further questions, the researcher contacted the participants by referring to them by phone calls or e-mail concurrent to data collection.

See Extended data: University Board’s interview and group discussion guide.

### Data analysis

The interview recordings were transcribed by the researchers. The content analysis with the inductive method was used for data analysis. ^17^ The researchers conducted a separate preliminary analysis of interview and group discussion transcripts. The data were read line by line, and key sentences and phrases were allocated, underlined, and coded. Similar codes were combined, and primary themes were made. Data reduction was conducted in all analyzed units until categories emerged, so that general, conceptive, and inductive data were put in the main theme. The researcher held meetings to edit and recode the themes in the process of data analysis. The meetings provided an opportunity to delineate, define themes, and discuss cultural and linguistic perspectives. The saturation of the data was reached after eleven interviews and six group discussions, but scheduled interviews and group discussions were completed and provided support for previous themes. The researchers continued the interactive process until an agreement was reached on the themes. The data of training records were analyzed simultaneously with the content analysis.

## Results

The participants were asked questions pertaining to the focus of the implementation of HAIs-PC education, the challenges of the faculty on teaching and students’ learning, facilities, and students' competency perceptions. The results indicated that there were challenges in HAIs-PC education in Vietnamese and Cambodian institutions. The challenges come from the curriculum, students’ and teachers’ characteristics, and universities. The diagnostic indicator results provided primary information on the designed curriculum regarding each academic year for the HAIs-PC module, contents, and credit hours for theory and practice. The interview and group discussion results showed that the challenges came from the implementation of the curriculum, an inactive learning environment, and unsupportive facilities at the university.

Most HEIs have been providing nursing education programs for bachelor students for more than ten years. These universities integrated HAIs-PC contents into the nursing curriculum during various study years. Mostly, nursing teachers provided lectures and lab practice of HAIs-PC to the nursing students in the second year of the study program. The students practiced providing care for patients in the hospitals in the third year of study, under the supervision of the clinical lecturers and nurses.

The total participants in the study were 49 managers, nursing faculty teachers, and clinical tutors. Vietnam (V) had 28 participants, with university 1 (U1) (16 participants), University 2 (U2) (12 participants), and Cambodia (C) had 21 participants, with university 3 (U3) (12 participants) and University 4 (U4) (9 participants). The detail is shown in Table 1.

**Table 1.** The participants of the study.

Country	HEI	Numbers of participants	Participants of each group (Gr 1: Gr 2: Gr 3)
**Vietnam**	University 1	16	3:9:4
	University 2	12	3:6:3
**Cambodia**	University 3	12	3:6:3
	University 4	9	3:3:3

In each HEI: VIs/CIs: Interview of Managers; VGs/CGs: Group discussion of Teachers and Clinical tutors.

*The diagnostic indicators of the curriculum*

The four universities offered a wide range of nursing degree levels. Universities in Vietnam had bachelor's degrees in nursing with large numbers of students, especially U1 currently has 1,733 nursing bachelor's students studying at the university. All universities in the countries lectured one to two credits for the HAIs-PC module, which, distributed time equally between theory and skill practice. In Asian countries, each credit corresponds to 15 hours of theory or 30 hours of skill practice. The institutions provided lecture and skill practice hours for HAIs-PC variously, U1 with 45 hours and U4 with 15 hours for the whole course. Clinical practice at the hospital for HAIs-PC was integrated into the other modules. All four universities provided students with knowledge and lab practice related to HAIs-PC. The contents were transmission routes and HAIs prevention measures; sterilization for and management of medical equipment; Sanitization of the hospital environment; Medical waste management; Prevention and control of blood and body fluid exposure and Infection surveillance. There was a slight difference in the program structure. A university in Vietnam arranged an HAIs-PC course with 3 credits, including a credit in microbiology and parasites HAIs-PC theory and clinical practice. The other universities provided the program with a different structure. The total hours of theory and practice ranged from 15 to 45 hours, in which, the practice hours were double compared to theory hours. Details are presented in Table 2.

**Table 2.** Indicators of the four higher education institutions and their nursing curriculum.

Characteristics	U1	U2	U3	U4
Number of academic years	4	4	4	4
Number of nursing students	1733	594	88	184
Numbers of lecturers	256	126	34	29
Numbers of tutors/mentors	80	17	6	8
Students/teachers ratio in lab practice	23	19	12	15
Number of HAIs PC module	1	1	1	1
Number of HAIs PC credits	2	2	1.5	1
Number of HAIs PC theory hours	15	15	15	10
Number of HAIs PC lab hours	30	30	15	5

*Results of current challenges of HAIs-PC education in the nursing curriculum in Asian countries*

**Figure 1.**
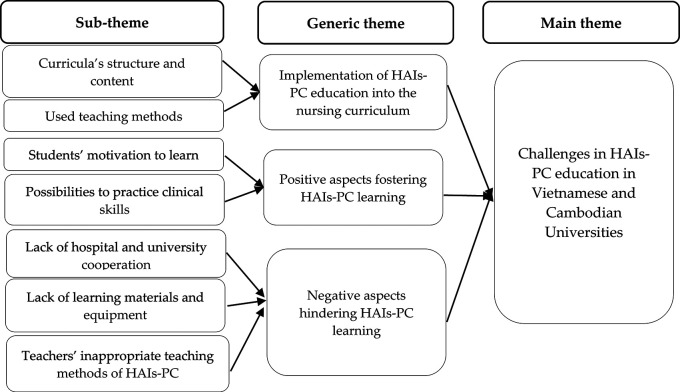
The challenges in HAIs-PC education in Vietnamese and Cambodian Universities.

The research findings were structured into three generic themes, which were the Implementation of HAIs-PC education into nursing curriculum, Positive aspects fostering IPC learning, and Negative aspects hindering HAIs-PC learning ( Figure 1).

*Implementation of HAIs-PC education into the nursing curriculum*

The generic theme was composed of sub-themes including ***Curricula’s* s *tructure and content* and *Used teaching methods***. The HAIs-PC education in the four institutions was recognized as an important aspect of nursing education. However, the ***Curricula’s structure and content*** in the institutions were different depending on the perception of the role of HAIs-PC in clinical settings. The participants indicated various perceptions regarding the current nursing curriculum in their universities. In both countries, the nursing curriculum contents, teaching materials, and teaching methods met the objectives related to the HAIs-PC course. The HEI regularly updated the structure and contents of HAIs-PC contents to meet the rapidly changing situation of the countries’ healthcare system and the needs for healthcare services. The participants indicated that continuous updating of the contents of the course always ensured a scientific and evidence-based curriculum before integrated teaching to students. Moreover, it seemed that the students gained the needed basic knowledge and skills on HAIs-PC during their learning process.

“ *Earlier the HAIs-PC unit focused only on the contents related to HAIs-PC, but now the unit is integrated with Microbiology and Parasitology. Therefore, this provides for students with an overview of the knowledge related to infection control” (VG2)*

A difficulty in the implementation of HAIs-PC education in the curricula was that the countries did not have clear enough guidelines from the Ministry of Health (MOH) and there was no common agreement between the academic and the hospital settings on HAIs-PC curriculum. Therefore, the Universities were not able to prioritize the corresponding HAIs-PC practical skills before students practiced in the hospital settings.

“ *We would like to have a specific guideline from the MOH regarding HAIs-PC training curricula that has consistency regarding the integration of knowledge and practice with clinical practice, and between the academic and clinical settings.* *” (CG2)*

However, the Covid-19 pandemic brought more consistency into the regular updating of teaching content met the need of healthcare service systems under the direction of MOH and WHO.

“ *Especially with the outbreak of the Covid-19 pandemic, we had updated the contents related to the HAIs-PC such as the use of personal protective clothing, cleaning, and disinfection*” *(VG1)*

“ *The lecturers participated and received certificates of open courses on HAIs-PC regarding Covid 19 by WHO at the request of the University to update knowledge for all students*”

***The used teaching methods*** were based on an assumption that nursing bachelor’s students need to gain enough knowledge and skills related to HAIs-PC while they study in classes and skill practice before they enter the clinical settings. Therefore, the universities designed a curriculum that provided bachelor students the lectures on basic knowledge and skill practice, in which the strategies and methods in teaching HAIs-PC were used to meet the requirements.

“ *Simulation method or teaching based on clinical situations is very important and necessary, it helps students connect knowledge and skills together as well as help students get closer to clinical reality*”

The lecturers have precepted the role of simulation in clinical teaching, especially in training students in positive thinking and decision-making skills for HAIs-PC. However, their capacity was insufficient to apply the method. In clinical settings, awareness of the exited inherent gap between the academic and clinical settings, the wards tried to create the best possible conditions to guide students while practicing HAIs-PC in the hospital.

*“Clinical experience and practice are very important to build simulation situations; some young teachers have difficulty implementing this teaching method” (VG1&2)*

*“…., new teaching methods need to be trained, … we have not been properly trained in simulation teaching methods … so it is difficult to apply” (CG1)*

### Positive aspects fostering HAIs-PC learning

The research results indicated that the Positive aspects fostering HAIs-PC learning were composed of the sub-themes of ***Students’ motivation to learn*** and the ***Possibilities to practice clinical skills***. Most of the universities informed that they tried to ***motivate students’ HAIs-PC learning*** by using different and more interactive teaching methods that promoted greater students’ participation. They also emphasized the need to combine different teaching methods to promote the integration of theory into practice. Moreover, dealing with the lack of practical equipment and tools, the University arranged extra-curricular laboratory rooms for students so that they had more opportunities to practice HAIs-PC skills.

*“We arrange instructor and senior students to support other students to practice on* HAIs-PC *skills at extra-curricular practice rooms” (CI1)*

Students were encouraged to participate in hospital quality improvement activities and research projects related to HAIs-PC in order to increase their interest in learning and enhance the student's experience in the module. Students, who conducted research or participated in data collection for a project, were awarded points for completing the study program at the University. Some universities considered that quality improvement activities and research projects on HAIs-PC should be emphasized in order to enhance the lecturers’ quality and encourage student participation.

“ *Many students participate in HAIs-PC research and quality improvement activities regarding HAIs-PC, students showed their innovative, creative thinking, this learning activity is quite helpful for students*”

The HEIs enhanced the ***possibilities to practice clinical skills*** related to HAIs-PC among students. The nursing curriculum in the countries does not include skill practice of HAIs-PC in clinical settings, which is integrated with other clinical modules. However, in recent years, a number of young lecturers were trained to use simulation pedagogy. Therefore, the Universities designed practical teaching using simulation pedagogy, with several of modern HAIs-PC equipment to increase students' practice opportunities.

*“Need to build a standard simulation to practice HAIs-PC. However, the standard simulation does not mean that we need to build a simulation in the nursing skill lab; we can integrate simulation into our hospital in which our students practice infection prevention and control*” *(VG1)*

### Negative aspects hindering HAIs-PC learning

The generic theme “negative aspects hindering HAIs-PC learning” was composed of the sub-themes ***Lack of hospital and university cooperation, Lack of learning materials and equipment*,** and ***Teachers’ inappropriate teaching methods of HAIs-PC.*** There was uncertainty among the leader and teacher groups about how HAIs-PC education was provided and if it was their responsibility. Many interviewed expressed uncertainties expressed as ***Lack of hospital and university cooperation**.* The managers indicated that the institutions had regular meetings with hospital leaders for implementing clinical training in the hospitals. Moreover, most of the institutions focused on enhancing the quality of teaching HAIs-PC in clinical settings. However, there was a number of factors affecting the quality of students' clinical skills training that they considered beyond their capabilities, e.g. challenges for learning when there were too many patients and students in the clinics.

“ *There are many students taking clinical placements at the same time in one ward leading to difficulty for tutors and lecturers to manage students in implementing HAIs-PC*” (CG2)

Some of the visiting lecturers, who worked at hospitals, stated having lack of pedagogical skills and theoretical knowledge of HAIs-PC education. Even though the universities had a training plan, the lecturers´ could not always attend the courses because of work overload at the hospital.

“ *Lack of uniformity in teaching method, and assessment method between tutors lead to students experiencing different wards; they receive different guidelines and assessment about HAIs-PC. These results in difficulty in identifying student weakness to assist students” (VG1&2)*

The results of the group discussion and interview indicated that the universities were ***lack of learning materials and equipment*** for HAIs-PC education **.** Hospital settings were very important in maintaining and developing students' HAIs-PC skills because students gradually improved their HAIs-PC competency during a period of clinical practice. The university always tried to invest in equipment. The lecturers taught practical skills by imagining the equipment because the equipment and infrastructure did not meet the requirement for practice. Many contents related to the practice of HAIs-PC were not taught in the skill labs. Therefore, the students could only observe these skills in the hospital such as clinical waste management, management of transmission routes, and sources of transmission.

*“Lack of equipment for teaching and practice of infection control is a problem … It is difficult to be able to equip a full set of equipment for HAIs-PC in academic settings because it is very costly. University should cooperate with the hospitals where HAIs-PC equipment is satisfactory so that students can experience”*

The university efforted to increase learning resources for students such as updating the textbooks every two years and increasing the books in the library. However, these efforts also failed to meet the current training needs of HEI, as quoted:

“ *Some of the textbook contents were outdated … Most students only use the required textbooks, rarely learn more from other resources …encounter problems with database access and language barriers” (VGs&CI1). “There is a lack of teaching tools for practice such as nursing procedure manuals or videos to assist students in self-studying” (VG2)*

Most of leader Boards and teachers indicated that the teachers currently used ***inappropriate teaching methods of HAIs-PC.*** The need to use different and more interactive teaching methods that promote greater participation with more active methodologies was highlighted. Teachers also emphasized the need to combine different teaching methods to enhance the competency of students in clinical settings. However, the application of these methods encountered difficulties. The capacity building of lecturers should have been focused on developing active learning and teaching skills such as case- and problem-based education. These methods could have helped students connect knowledge and skills together as well as help students get closer to clinical reality. Currently, in the HEI, there was a gap between theory and practice in HAIs-PC education, as students did not have a chance to practice with the functioning equipment.

*“Lecturers mainly provided students with purely theoretical knowledge…while practicing, students performed procedures using the checklist with few real clinical situations or simulation scenarios … students shown less critical thinking, problem-solving and teamwork skills after completing the course” (CGs & VG1)*

The students performed well on the basic skills of HAIs-PC in the skill lab, however, while taking care of real patients in the clinical settings, students sometimes became confused. The students had difficulty in making decisions and following technical procedures as well as the principles of HAIs-PC correctly. The reason was that students were not familiar with clinical situations before practicing in the hospitals but were just taught single practical skills. The positive learning skills, such as critical thinking, problem-solving, and teamwork, were emphasized only in theory classes instead of skill practice. Therefore, simulations may be a good teaching method in the future in Asian countries, because it helps to combine a variety of cognitive, psychomotor, and emotional skills.

*“When asking students to do single skills such as hand hygiene or personal protective equipment, students do the right thing and are quite proficient but when put in a specific situation and patients, students sometimes forget to perform it or performed incorrectly” (CG2)*

Although, the contents and practice of HAIs-PC were integrated into clinical-related modules. The clinical teachers and tutors were not confident in their integrated knowledge of HAIs-PC, then they rarely strengthen the HAIs-PC practice in clinical settings.

“ *Having many nursing subjects applying the principles of HAIs-PC in the delivery of nursing care, for example, fundamental of nursing, medical-surgical nursing clinical practice, however, the instructors rarely further reinforced IPC skills while practicing in hospitals” (CG1)*

The students lacked the initiative to apply HAIs-PC skills or avoided or missed out on safety awareness in providing care to the patients in clinical settings. The leaders attributed the cause to the traditional culture of providing a passive learning environment for students. Moreover, the large number of students in theory and practice classes weakened lecturers´ possibilities to reach all the learning outcomes. Furthermore, lecturers should be retrained periodically to update their HAIs-PC expertise to become more confident in the teaching methods. Updating this knowledge also helps lecturers at institutions have uniformity in teaching methods.

“ *Many nursing teachers do not feel confident in teaching the theory of infection and prevention subject because they have not regularly received training on infection and prevention subject” (VG2)*

“ *We made great efforts to build an e-learning course on HAIs-PC to promote autonomy, self-study, and problem-solving for learners. The relevance and applicability of the learned knowledge about IPC at the University in the clinical settings was a common concern”* (VI2)

“ *A practice group can be up to 20 to 25 students; this is very difficult to manage whether students achieve other learning objectives set out in the lesson or not” (VG2)*

## Discussion

This study explored the challenges of HAIs-PC education in nursing curriculum in two Vietnamese and two Cambodian HEIs. The exploratory study was conducted in two Vietnam and two Cambodia HEIs with 49 participants who were leaders, nursing teachers, and clinical tutors. There was diversity in the ratio of students to faculty between universities and between countries, specifically, the ratio in a Vietnam university was 22.7 while that at Cambodia university was 12.0. The research results indicated that there were various hours in theory and laboratory practice from university to university. Furthermore, there were differences in the hours per credit requirement between the two countries, particularly with regard to the number of hours per credit for practical courses. The results correspond to the results of previous studies on the diversity in the regulation system of nursing professional training of two countries. ^18^^,^^19^ The qualitative results emerged the main theme challenges in HAIs-PC education with generic themes of Implementation of HAIs-PC education into nursing curriculum, Positive aspects fostering HAIs-PC learning, and Negative aspects hindering HAIs-PC learning.

Research results on the positive aspects of HAIs-PC education at the HEIs included motivating students’ learning and enhancing students' ability to practice. HEIs strived to apply active teaching methods to enhance student engagement in learning. The addition of an extra-curricular skill practice for students was also a positive method to be applied. In addition, the case-based pedagogy had begun to improve students' practical capacity on HAIs-PC. The research results were similar to previous studies to enhance the learning and practice of HAIs-PC of nursing students. ^20^^,^^21^ Kim and colleagues (2020) indicated that simulation pedagogy improved HAIs-PC competence by increasing knowledge, compliance, and practical quality and outcomes in patients. ^22^

The negative aspects hindering HAIs-PC learning were revealed by the managers, lecturers, and clinical tutors. The participants admitted that they used active teaching methods in delivering HAIs-PC lectures, but it was not effective. These perceptions were consistent with nursing instructors in some Asian countries because they were familiar with traditional teaching methods, along with a large number of students in study groups. ^23^^,^^24^ The connection, application of knowledge, and practice between the University and the clinical environment was also a challenge to the teaching capacity of lecturers. Therefore, improvements are urgently needed in HAIs-PC education for nursing bachelors at Asian universities. To create a positive learning environment, specifically related to HAIs-PC education, efforts need to come from teachers, students, and especially from educational institutions. Therefore, in order to improve the challenges in HAIs-PC education for nursing students, HEIs of Vietnam and Cambodia should pay attention to the following changes and improvements. Firstly, the universities should build an active learning model in which increased content knowledge, critical thinking, problem-solving abilities, and positive attitudes toward learning and make a fluency between theory and clinical practice. ^25^ Although leaders and teachers claimed that they regularly updated the books, however, updating should focus on making the books more appealing to learners. Instead of simply providing content, the books should include images and interesting real-life cases related to HAIs-PC. This may make an interesting first impression of HAIs-PC learning. Moreover, the orientation session is equally important to help students understand the significance of HAIs-PC learning in their professional practice. In theory, the teachers should use active teaching methods, create two-way interaction and initiative in learning, and provide timely two-way feedback. For practice, the simulation pedagogy would provide students with the opportunity to familiarize themselves with and experience HAIs-PC before practicing in real clinical settings. ^26^

Unsupported facilities for HAIs-PC learning included the lack of equipment, teaching materials, and clinical facilities. This result corresponds to some previous studies. ^27^^,^^28^ Through the nursing education system, lecturers and instructors should be empowered and encouraged to provide an enhanced nursing education approach that applies local facilities and resources, as well as enhance awareness of local resources. Developing countries’ universities should adopt and adapt active teaching methods to improve nursing education by utilizing available resources. ^29^^,^^30^ The use of different active learning methods, especially, simulation pedagogy may improve communication and critical thinking skills and enhance safety in healthcare for the patients and nursing students themselves. ^31^

The research results indicated that one of the challenges was the lack of knowledgeable and skilful lecturers in the field of HAIs-PC education. Many nursing teachers were not confident in teaching theory and practice on HAIs-PC because they have not been regularly trained on the module. The results were congruent with previous studies that the teachers need to be more competent by attending regular training or workshops on HAIs-PC education. ^32^ The InovSafeCare Pedagogical Model for HAIs-PC education was implemented and presented the effectiveness of student HAIs-PC outcomes in Europe. ^33^ The model focuses on the comprehensive application of active teaching pedagogies to theoretical, practical, and clinical learning environments. In particular, the simulation method with well-designed scenarios based on clinical reality enhanced students’ ability to critically thinking and problem-solving HAIs-PC problems. ^34^ A similar culturally adapted model should be piloted to evaluate the model on HAIs-PC students’ outcomes. Moreover, the universities should have regular training on HAIs-PC for the teachers, as well as assign them as clinical instructors in the HAIs-PC department at the hospital for their updating knowledge and skills. Besides supplementing competency related to HAIs-PC, lecturers also need to be trained to improve their pedagogical skills as well as how to deploy active teaching methods, especially simulation teaching. In addition, the capacity building of evidence-based research and practice in the field of HAIs-PC should also be considered to enhance students’ and lecturers’ competency in HAIs-PC. ^35^^,^^26^

The present study used a qualitative design with in-depth interviews and focus group discussions on diverse participants and a quantitative design to ensure various perspectives were obtained from the participants. In addition, the number of focus groups and in-depth interviews was continuously conducted until the final interview and discussion, although the researcher found the data were saturated, which made the research results identified rich and reliable. However, only data from leader boards, teachers, and tutors were collected, without perceptions from students. Future research should be explored these challenges from the student’s perspective for a holistic perspective on the issue.

The research results identified that challenges in HAIs-PC education were considered sensitive topics for their HEIs. However, face-to-face interviews and focus group discussions with participants may create the impression that participants could be trying to “hide” real challenges or overemphasize the positive aspects of their HEIs. During the interviews and focus group discussions, the researcher emphasized that the research purpose was to identify common challenges, not problems with the participants themselves, therefore, reducing concealment. In addition, interviews and focus group discussions conducted in universities had various characteristics and cultures, the research results may be generalized to the other HEIs in Asian countries.

## Conclusions

The study may provide evidence of the challenges to the current HAIs-PC education in the nursing curriculum in Vietnam and Cambodia. The negative aspects hindering HAIs-PC education of inappropriate pedagogy, equipment and materials, and university and hospital cooperation were the challenges. The evidence may suggest useful solutions for future nursing education on HAIs-PC practice, consequently reducing the prevalence of HAIs in Vietnam and Cambodia.

Providing a robust, updated HAIs-PC education curriculum that is culturally consistent and contextually relevant is an important first step in improving nursing education outcomes and quality care. The faculty and clinical instructors, who provide direct education to nursing students, are ideally positioned to develop students' HAIs-PC skills for future nurses with safe and effective care competency. Regularly updating content, applying active teaching methods in skills training, and utilizing existing local resources may improve the effectiveness of HAIs-PC education for nursing students.

## Disclaimer

The European Commission's support for the production of this publication does not constitute an endorsement of the contents, which reflect the views only of the authors, and the Commission cannot be held responsible for any use which may be made of the information contained therein.

## Data Availability

Figshare: Current challenges in teaching HAIs-PC in nursing education in Vietnam and Cambodia: a qualitative study. https://doi.org/10.6084/m9.figshare.23669277.v2. ^36^ This project contains the following underlying data:
- HAIs-PC data The qualitative data underlying this study are available on request from the corresponding author. The data are not publicly available due to ethical considerations regarding personal information and to respect what was written in the signed informed consent. Figshare: Current challenges in teaching HAIs-PC in nursing education in Vietnam and Cambodia: a qualitative study. https://doi.org/10.6084/m9.figshare.23669277.v2. ^36^ This project contains the following extended data:
• 26-items record collection instrument • The University Board’s interview guide • The lecturer focus group topic guide • The tutor focus group topic guide Data are available under the terms of the Creative Commons Attribution 4.0 International license (CC-BY 4.0).

## PrevInf Group

The authors would like to thank all the leader Boards, nursing faculty, clinical instructors involved in this study. The authors would also like to thank Erasmus+ for the ongoing support with the project activities. All authors have given their permission for their names and affiliations to be included in this publication. The following contributors opted to have their names acknowledged:

PrevInf Group: Nam Dinh University of Nursing - Vietnam, Savonia University of Applied Sciences - Finland, Nursing School of Coimbra (ESEnfC) – Portugal, Centre for Innovative Biomedicine and Biotechnology – Portugal, Hai Duong Medical Technical University - Vietnam, International University - Cambodia, Bolyno Institute – Cambodia.

Vu Van Thanh, Nam Dinh University of Nursing, Vietnam. ID: 0009-0009-4531-8657. Contribution(s): Visualization, project administration.

Ngo Huy Hoang, Nam Dinh University of Nursing, Vietnam. ID: 0000-0002-5895-3352. Contribution(s): Visualization, supervision, project administration.

Mai Thi Lan Anh, Nam Dinh University of Nursing, Vietnam. ID: 0000-0003-2487-7766. Contribution(s): Methodology, software, validation, formal analysis, investigation, writing original draft preparation, writing review and editing, supervision, project administration.

Vu Thi La, Nam Dinh University of Nursing, Vietnam. ID: 0009-0009-7635-6461. Contribution(s): Validation, formal analysis, investigation, supervision.

Hoang Thi Minh Thai, Nam Dinh University of Nursing, Vietnam. ID: 0000-0002-5614-5124. Contribution(s): validation, formal analysis, investigation.

Nguyen Thi Thanh Huong, Nam Dinh University of Nursing, Vietnam. ID: 0000-0002-6860-2521. Contribution(s): validation, formal analysis, investigation.

Pham Thi Bich Ngoc, Nam Dinh University of Nursing, Vietnam. ID: 0000-0003-2676-8854. Contribution(s): Validation, Investigation.

Le Van Cuong, Nam Dinh University of Nursing, Vietnam. ID: 0009-0007-7445-2631. Contribution(s): Investigation.

Mai Thi Thanh Thu, Nam Dinh University of Nursing, Vietnam. ID: 0000-0002-9556-7688. Contribution(s): Validation, formal analysis.

Pham Thi Thuy Chinh, Nam Dinh University of Nursing, Vietnam. ID: 0000-0003-2640-5578. Contribution(s): Formal analysis, investigation.

Marja Silén-Lipponen, Savonia University of Applied Sciences, Finland. ID: 0000-0001-6452-2447. Contribution(s): Conceptualization, methodology, validation, formal analysis, writing original draft preparation, writing review and editing, visualization, supervision.

Leena Koponen, Savonia University of Applied Sciences, Finland. ID: 0009-0004-0213-1263. Contribution(s): Conceptualization, methodology, validation, formal analysis, writing original draft preparation, writing review and editing, visualization, supervision.

Ulla Korhonen, Savonia University of Applied Sciences, Finland. ID: 0000-0003-1632-3712. Contribution(s): Validation, formal analysis.

Mikko Myllymäki, Savonia University of Applied Sciences, Finland. ID: 0009-0001-5352-0013. Contribution(s): Validation, formal analysis.

Pedro Parreira, Escola Superior De Enfermagem De Coimbra, Portugal. ID: 0000-0002-3880-6590. Contribution(s): Conceptualization, methodology, resources, writing review and editing, supervision, project administration.

Anabela Salgueiro-Oliveira, Escola Superior De Enfermagem De Coimbra, Portugal. ID: 0000-0002-8231-8279. Contribution(s): Writing review and editing, supervision, project administration.

Lurdes Lomba, Escola Superior De Enfermagem De Coimbra, Portugal. ID: 0000-0003-1505-5496. Contribution(s): Supervision, project administration.

Paulo Santos-Costa, Escola Superior De Enfermagem De Coimbra, Portugal. ID: 0000-0003-0761-6548. Contribution(s): Writing review and editing, supervision, project administration.

Filipe Paiva-Santos, Escola Superior De Enfermagem De Coimbra, Portugal. ID: 0000-0003-0962-6635. Contribution(s): Writing review and editing, supervision, project administration.

João Pardal, Escola Superior De Enfermagem De Coimbra, Portugal. ID: 0000-0001-8162-4808. Contribution(s): Conceptualization, methodology, resources, writing review and editing, supervision, project administration.

Dinh Thi Xuyen, Hai Duong Medical Technical University, Vietnam. ID: 0000-0002-9001-5681. Contribution(s): Supervision, project administration.

Do Thi Thu Hien, Hai Duong Medical Technical University, Vietnam. ID: 0000-0002-9428-5099. Contribution(s): Validation, formal analysis, investigation, supervision.

Pham Thi Thanh Phuong, Hai Duong Medical Technical University, Vietnam. ID: 0009-0008-9607-5862. Contribution(s): Validation, formal analysis, investigation.

Nguyen Thi Hue, Hai Duong Medical Technical University, Vietnam. ID: 0009-0003-3063-7395. Contribution(s): Validation, investigation.

Vu Thi Hai, Hai Duong Medical Technical University, Vietnam. ID: 0009-0005-6367-9756. Contribution(s): Investigation.

Dam Thi Thuy, Hai Duong Medical Technical University, Vietnam. ID: 0000-0002-7456-7662. Contribution(s): Validation, investigation.

Dinh Thi Dieu Hang, Hai Duong Medical Technical University, Vietnam. ID: 0000-0002-4143-1419. Contribution(s): Supervision, project administration.

Pham Thi Cam Hung, Hai Duong Medical Technical University, Vietnam. ID: 0000-0002-0712-6058. Contribution(s): Supervision, project administration.

Do Thi Hue, Hai Duong Medical Technical University, Vietnam. ID: 0009-0008-5069-3024. Contribution(s): Validation, formal analysis, investigation.

Vu Thi Quyen, Hai Duong Medical Technical University, Vietnam. ID: 0009-0008-8886-938X. Contribution(s): Formal analysis, investigation.

Nget Manndy, International University, Cambodia. ID: 0000-0002-5787-2585. Contribution(s): Validation, formal analysis, investigation, writing review and editing.

Neth Barom, International University, Cambodia. Contribution(s): Validation, formal analysis, investigation, writing review and editing, supervision.

Vouch Phisith, International University, Cambodia. Contribution(s): Validation, investigation.

Gnan Channoeun, International University, Cambodia. Contribution(s): Project administration.

Chhay Kry, International University, Cambodia. Contribution(s): Validation, formal analysis, investigation.

Y Sokchhay, International University, Cambodia. Contribution(s): Validation, formal analysis, investigation, supervision.

Siv Sarin, Bolyno Institute, Cambodia. Contribution(s): Validation, formal analysis.

Im Soresreyrath, Bolyno Institute, Cambodia. Contribution(s): Validation, formal analysis.

Ouch Bory, Bolyno Institute, Cambodia. Contribution(s): Validation, formal analysis.

Sek Sophon, Bolyno Institute, Cambodia. Contribution(s): Validation, formal analysis.

Mao Eam, Bolyno Institute, Cambodia. Contribution(s): Validation, formal analysis.

Vannarith Nhem, Bolyno Institute, Cambodia. Contribution(s): Validation, formal analysis.

Song Chhiay, Bolyno Institute, Cambodia. Contribution(s): Validation, formal analysis.

Heng Socheat, Bolyno Institute, Cambodia. Contribution(s): Validation, formal analysis.

Em Sovannarith, Bolyno Institute, Cambodia. Contribution(s): Validation, formal analysis.

Pao Panha, Bolyno Institute, Cambodia. Contribution(s): Validation, formal analysis.

João Graveto, Escola Superior De Enfermagem De Coimbra, Portugal. ID: 0000-0001-6974-9421. Contribution(s): Conceptualization, methodology, resources, writing review and editing, supervision, project administration.
