# Supplementary material for: Current challenges in teaching healthcare-associated infections prevention and control in nursing education in Vietnam and Cambodia: a qualitative study
Source: F1000Res. 2024 Jul 30;12:1535. Originally published 2023 Nov 30. [Version 2] doi: 10.12688/f1000research.139734.2 (PMC11325131; doi:10.12688/f1000research.139734.2)
Supplement: Peer review history

# Reviewer report: Reviewer response for version 2

**DOI:** 10.5256/f1000research.169674.r312882

**Published:** 2024-09-04

**Authors:** Ruijie Yin

**Affiliations:** 1 MedStar Health (Ringgold ID: 2613), Washington, DC, USA; 2 University of Miami, Miami, USA

This study aimed to identify challenges in teaching healthcare-associated infection prevention and control (HAIs-PC) to nursing students in Vietnam and Cambodia. Through interviews and focus groups, three main themes emerged: implementation challenges, positive factors fostering learning, and negative factors hindering learning. The findings highlight the need for universities in these countries to develop more effective teaching methods for HAIs-PC education to enhance student outcomes and improve patient safety. The revised version of this manuscript has addressed the comments from other reviewers, I do not have more comments for this article.

Is the work clearly and accurately presented and does it cite the current literature?

Yes

If applicable, is the statistical analysis and its interpretation appropriate?

Yes

Are all the source data underlying the results available to ensure full reproducibility?

Yes

Is the study design appropriate and is the work technically sound?

Yes

Are the conclusions drawn adequately supported by the results?

Yes

Are sufficient details of methods and analysis provided to allow replication by others?

Yes

Reviewer Expertise:

Healthcare-associated infections.

I confirm that I have read this submission and believe that I have an appropriate level of expertise to confirm that it is of an acceptable scientific standard.

# Reviewer report: Reviewer response for version 2

**DOI:** 10.5256/f1000research.169674.r308858

**Published:** 2024-08-14

**Authors:** Jaouad El-Hilaly

**Affiliations:** 1 Sidi Mohamed Ben Abdellah University,, Fez,, Morocco

The authors have successfully responded to my concerns, subsequently,  I have no more comments. Therefore, I presume the article has been significantly improved and deserves to be indexed in F1000 Research Journal.

Is the work clearly and accurately presented and does it cite the current literature?

Yes

If applicable, is the statistical analysis and its interpretation appropriate?

Not applicable

Are all the source data underlying the results available to ensure full reproducibility?

No

Is the study design appropriate and is the work technically sound?

Partly

Are the conclusions drawn adequately supported by the results?

Partly

Are sufficient details of methods and analysis provided to allow replication by others?

No

Reviewer Expertise:

Nursing Education; Therapeutic education, Teachers training, Psychometrics, Physiology and Pharmacology

I confirm that I have read this submission and believe that I have an appropriate level of expertise to confirm that it is of an acceptable scientific standard.

# Reviewer report: Reviewer response for version 1

**DOI:** 10.5256/f1000research.153033.r276830

**Published:** 2024-05-25

**Authors:** Jaouad El-Hilaly

**Affiliations:** 1 Sidi Mohamed Ben Abdellah University,, Fez,, Morocco

In qualitative research, data triangulation through FGs and IDI interviews may lead to a more comprehensive knowledge of the topic of interest. The authors have an acceptable Data source triangulation, they collect data through interviews with individuals (IDI interviews) or groups (FGs). However, some issues need addressing to validate the trustworthiness of findings:

  

1. The number of participants of each FG has been mentioned in Table 1, but there is no information about the participants interviewed individually.
2. The data source of both methods should be reported distinctly.
3. How do both methods (IDIs and FGs) lead to the convergence of the data?
4. to understand how various types of data contribute to understanding of phenomena
5. How does the combined data enhance understanding of the current issue, and answer the research question?
6. Compare the results of both methods of data collection (IDIs and FGs)
7. HAC-PC should be defined in the abstract.
8. Remove the acronym in the title if the submission guidelines do not permit it.
9. Thematic Analysis Software is not reported.
10. What are the limitations of this research paper?

Is the work clearly and accurately presented and does it cite the current literature?

Yes

If applicable, is the statistical analysis and its interpretation appropriate?

Not applicable

Are all the source data underlying the results available to ensure full reproducibility?

No

Is the study design appropriate and is the work technically sound?

Partly

Are the conclusions drawn adequately supported by the results?

Partly

Are sufficient details of methods and analysis provided to allow replication by others?

No

Reviewer Expertise:

Nursing Education; Therapeutic education, Teachers training, Psychometrics, Physiology and Pharmacology

I confirm that I have read this submission and believe that I have an appropriate level of expertise to confirm that it is of an acceptable scientific standard, however I have significant reservations, as outlined above.

# Response

**Published:** 2024-07-02

**Authors:** Truong Tuan Anh

**Affiliations:** Nursing, Nam Dinh University of Nursing, Nam Dinh city, Vietnam

Thanks for the valuable comments. Kindly find the responses to your comments below,

 1. The number of participants of each FG has been mentioned in Table 1, but there is no information about the participants interviewed individually.

 Response: 49 people met the requirements to participate in the study, of which 12 managers participated in individual in-depth interviews. At the same time, 8 group discussions were conducted with 37 lecturers and clinical instructors.

 2. The data source of both methods should be reported distinctly.

 Response: All in-depth interviews and group discussions were anonymous, and each participant was given a code. The interviewers recorded and transcribed data throughout the interviews. The researchers conducted total of 12 in-depth interviews with 12 University Board members and 8 group discussions from lecturers and tutors (4 groups of lecturers and 4 groups of tutors).

 3&4. How do both methods (IDIs and FGs) lead to the convergence of the data? to understand how various types of data contribute to the understanding of phenomena.

 Response: We added a description in the discussion to explain for the importance of various data to contribute to understanding of phenomena.

 5. How does the combined data enhance understanding of the current issue, and answer the research question?

 Response: The present study used a qualitative design with in-depth interviews and focus group discussions on diverse participants and a quantitative design to ensure various perspectives were obtained from the participants. In addition, the number of focus groups and in-depth interviews was continuously conducted until the final interview and discussion, although the researcher found the data were saturated, which made the research results identified rich and reliable. The combined data provided a comprehensive insight into the prevailing challenges of HAIs-PC education in nursing curricula across Asian countries. By analyzing these diverse data sources, researchers could gain a nuanced understanding of the multifaceted issues surrounding HAIs-PC education, including curriculum effectiveness, educational strategies, and barriers to learning. The quantitative data offered statistical evidence regarding the prevalence and impact of HAIs, while deep interviews and focus group discussions facilitated the exploration of nuanced perspectives and experiences of educators and students. This integrated approach not only elucidated the current state of HAIs-PC education but also enabled researchers to address the research question by identifying key areas for improvement and informing evidence-based interventions to enhance IPC learning outcomes in nursing education within Asian contexts.

 6. Compare the results of both methods of data collection (IDIs and FGs)

 Response: The results obtained from both methods of data collection, namely deep interviews conducted with The University Board and focus group discussions with lecturers and tutors regarding their perceptions of the Healthcare-Associated Infections Prevention and Control (HAIs-PC) program in their nursing curriculum, offered complementary insights into the effectiveness and challenges of the program implementation. Deep interviews with The University Board members provided a high-level perspective on the overarching goals, strategic planning, and institutional support for the HAIs-PC program. These interviews may yield insights into the alignment of the program with broader educational objectives and institutional priorities. On the other hand, focus group discussions with lecturers and tutors delved into the frontline experiences, perceptions, and challenges faced in the delivery of the HAIs-PC program. These discussions overall offered a granular understanding of the practical implementation issues, pedagogical strategies, and student engagement within the program. By combining the results of both methods, researchers can triangulate findings, identify converging themes, and gain a comprehensive understanding of the HAIs-PC program's strengths and weaknesses from both strategic and operational perspectives.

 7. HAI-PC should be defined in the abstract.

 Response: Added a definition of HAIs-PC in the abstract

 8. Remove the acronym in the title if the submission guidelines do not permit it.

 Response: Removed the acronym in the title. Title: Current Challenges in Teaching Healthcare-associated Infections Prevention and Control in Nursing Education in Vietnam and Cambodia: A Qualitative Study

 9. Thematic Analysis Software is not reported.

 Response: The content analysis with the inductive method was used for data analysis. ^17^ The researchers conducted a separate preliminary analysis of interview and group discussion transcripts. The data analysis process was supported by Nvivo 11 software (QSR International) for facilitating the qualitative content analysis.

 10. What are the limitations of this research paper?

 Response: However, the study has some limitations. Firstly, only data from leader boards, teachers, and tutors were collected, without perceptions from students. Future research should be explored these challenges from the student’s perspective for a holistic perspective on the issue.

 Secondly, the research results identified that challenges in HAIs-PC education were considered sensitive topics for their HEIs. However, face-to-face interviews and focus group discussions with participants may create the impression that participants could be trying to “hide” real challenges or overemphasize the positive aspects of their HEIs. During the interviews and focus group discussions, the researcher emphasized that the research purpose was to identify common challenges, not problems with the participants themselves, therefore, reducing concealment. In addition, interviews and focus group discussions conducted in universities had various characteristics and cultures, the research results may be generalized to the other HEIs in Asian countries.

# Reviewer report: Reviewer response for version 1

**DOI:** 10.5256/f1000research.153033.r276829

**Published:** 2024-05-14

**Authors:** Khin Thandar Aung

**Affiliations:** 1 Kulliyyah of Nursing, International Islamic University, Kuala Lumpur, Malaysia

The study emphasises the significant challenges in HAIs-PC education in Asian higher education institutions. To improve professional safety and enhance practice outcomes, universities need to address these challenges by implementing suitable teaching methods and ensuring sufficient resources for practical training. Future research should concentrate on evaluating the effectiveness of interventions that aim to overcome these barriers and improve HAIs-PC education in nursing curricula.

Reviewer Expertise:

nursing education, e-learning, MOOC course, emergency nursing, oncology nursing, public health emergency,

I confirm that I have read this submission and believe that I have an appropriate level of expertise to confirm that it is of an acceptable scientific standard.
